# Prevalence of Torque Teno Virus in Portuguese Children Younger Than 3 Years Old: A Single-Center Study

**DOI:** 10.1177/00099228251339047

**Published:** 2025-05-09

**Authors:** Maria-Jesus Chasqueira, Pedro Paixão, Carla Barreiros, Madalena Tuna, Mónica Marçal, Paulo Paixão

**Affiliations:** 1 Comprehensive Health Research Centre, NOVA Medical School - Faculdade de Ciências Médicas, Universidade NOVA de Lisboa, Lisboa, Portugal; 2 NOVA Medical School - Faculdade de Ciências Médicas, Universidade NOVA de Lisboa, Lisboa, Portugal; 3 Unidade de Neonatologia-Serviço de Pediatria, Hospital São Francisco Xavier CHLO, Estrada Do Forte Do Alto Do Duque, Lisboa, Portugal

**Keywords:** Torque Teno virus, pediatric population, saliva, plasma, real-time PCR

## Abstract

The objective of this study was to determine the prevalence of Torque Teno virus (TTV) infection in Portuguese children younger than 3 years old and to compare its detection in blood *versus* saliva. Samples, 242 saliva and 20 plasma, were collected from neonates and children. A real-time polymerase chain reaction was applied to detect the TTV load. Eighteen paired samples of saliva and plasma were concordant for viral DNA, and a correlation between both was statistically significant. The prevalence of TTV was 75.5% in children and 3.6% in neonates, but it was not possible to determine the origin of positive cases in neonates, if they were true cases acquired before or after birth. Overall, the results suggest that TTV transmission occurs mainly in early childhood. Despite the small sample size, the good saliva/plasma correlation obtained is promising, but larger studies are needed to validate saliva samples for epidemiological studies for TV.

## Introduction

Torque teno virus (TTV) is a single-stranded circular DNA virus, detected for the first time in a Japanese patient in 1997. This virus belongs to the genus Alphatorquevirus of the Anelloviridae family and constitutes a vast group of viruses related to each other, but with a high degree of genetic variability.^1^ This virus has a high worldwide prevalence, over 70%, depending on the sensitivity of the method used, as well as the geographical area.^2,3^

So far, TTV has not been considered pathogenic. Some potential associations with some pathologies have been described, but the evidence that TTV is directly responsible for a pathologic state is lacking.^4^ However, its relationship with the immune system is already well established, namely the increase in viral loads with low levels of cellular immunity, especially in transplant patients.^5^

It has been recently revealed that TTV is transmitted in early childhood,^6-8^ although the exact transmission mechanisms remain undescribed. Previous reports conclude that intrauterine infection is unlikely, as TTV has not been detected in umbilical cord blood or neonate blood collected 1 week after birth, which emphasizes perinatal infection as a more probable transmission mechanism.^8,9^ Subsequently, TTV transmission can occur in infancy, initially by contact with the mother, and later through increased social contacts (schools and other family members).^2^

Although blood is considered the reference sample for the detection of TTV, its collection for epidemiological purposes is difficult, particularly in studies done on children. Saliva can be considered a promising alternative to blood, given its easier collection, with several studies indicating a higher prevalence of TTV in saliva samples than in blood.^7,10,11^

To the best of our knowledge, TTV prevalence in Portugal has only been described in adults,^12^ hence the prevalence in children remains uncharacterized. Therefore, the primary objective of the present study is to evaluate the TTV prevalence in a Portuguese pediatric population in the first 3 years of life, through virus detection in saliva samples, aiming to see if TTV transmission occurs mainly during early childhood. Second, this study aimed to assess the concordance of TTV DNA in the paired saliva and plasma samples to explore the possibility of using saliva for epidemiological studies with this virus.

## Methods

### Patients

Two hundred and forty-two children were included in this study, of which 136 were neonates admitted to the nursery of the Neonatology Unit in a Hospital in the Lisbon area (all the samples in this group were collected in the first week of life. Mean age: 2 days. Male/female: 60/76). The neonates were recruited from the nursery, and inclusion criteria were that their condition was stable and they could remain with their mothers. All were born after 35 weeks (8 of them were premature babies born between 35 and 37 weeks, the rest after 37 weeks). Eleven had low birth weight (<2500 g), 5 of them were premature. Thirty-seven were born by cesarean section, 4 by forceps, 20 by ventouse suction cup, and the rest had a normal delivery. Premature babies, neonates fed by nasogastric tube, and those with comorbidities were excluded.

The other 106 were children aged between 1 and 3 years (Mean age: 27 months. Male/female: 57/49), who went to the Pediatric Emergency Department of the same Hospital. All had mild acute pediatric infectious disease, and severe cases (which required hospitalization), and children with immunosuppression or comorbidities were excluded (the information was obtained through interviews with parents). This choice was due to opportunity, as obtaining blood samples from children in the Emergency Department is easier. The first 20 children (Mean age: 20.3 months. Male/female: 14/6) whose parents authorized the use of blood for the determination of plasma viral load were selected for the comparison study between saliva and plasma samples. Both samples were collected simultaneously to correlate the TTV viral load in the 2 biological samples.

This study was approved by the Centro Hospitalar Research Ethics Committee and followed the ethical standards set by the Declaration of Helsinki. Participation in the survey required parental authorization and the obtaining of signed informed consent from all children.

### Samples

Samples were collected between October 2018 and June 2019. Collections were performed at the nursery of the Neonatology Unit and the Pediatric Emergency Department. Saliva samples were collected with a sterile Rayon swab (F.L. Medical, Torreglia, Italy), introduced into the oral cavity for 5 seconds, and, then placed in a CLEAR Line tube (CryoGen), with 500 µL of RPMI 1640 medium (Life Technologies, Paisley, UK)). Blood samples were collected in blood count tubes (S-Monovete, Sarstedt, Germany).

After collection, the samples were stored at 2°C to 4°C and transported within 24 hours to the Laboratory of Microbiology of the NOVA Medical School (NMS).

### Sample Processing

DNA extraction was performed with PureLink Genomic DNA Minikit (Invitrogen, Carlsbad, California) for all samples, following the manufacturer’s instructions. A quantitative in-house (real-time) polymerase chain reaction targeting a highly conserved segment of the UTR, was performed on the Applied Biosystems 7500 Fast Real-Time PCR System (Applied Biosystems, Foster City, California). Briefly, a segment of 63 nucleotides was amplified with the primers AMTS_Fw (5ʹ-GTG CCG IAG GTG AGT TTA-3ʹ), AMTAS_Rv (5ʹ-AGC CCG GCC AGT CC-3ʹ), and probe FAM5ʹ-TCA AGG GGC AAT TCG GGC T-3ʹTAMRA, in a final reaction volume of 25 µL (of which 5 µL was DNA sample).^13^ A plasmid (pCR2.1-TTV-TA278_81-280) was used as a quantification standard.

All reactions were performed in duplicate, and, in addition, internal control was performed, by adding 2 µL of plasmid pCR2.1-TTV-TA278_81-280 at a concentration of 5.3 log_10_ copies/mL to each sample.

### Statistical Analysis

Pearson correlation coefficient was used to analyze the relation between viral loads in saliva and plasma samples. Statistical significance was considered when *P* value <.05. Data analysis was conducted on SPSS version 20.0 (IBM, Armonk, New York).

## Results

**Figure 1.**
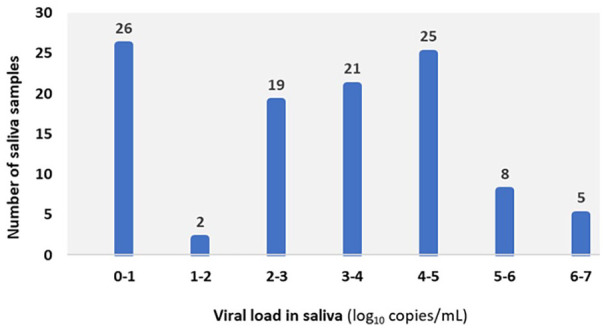
Distribution of TTV viral loads in saliva samples from children aged 1 to 3 years.

Of the 136 saliva samples from neonates, 5 (3.7%) were positive for TTV DNA, and the remaining 131 (96.3%) were negative. The median viral load of the positive samples was 2.32 log_10_ copies/mL, ranging between 2.22 log_10_ copies/mL and 2.88 log_10_ copies/mL (Figure 1).

In the group of children aged 1 to 3 years, 75.5% (80/106) were positive and 24.5% (26/106) were negative for TTV DNA. The viral loads obtained, with a median of 3.89 log_10_ copies/mL, ranged from 1.49 log_10_ copies/mL to 6.38 log_10_ copies/mL (Figure 1).

Regarding the paired samples of plasma and saliva collected, the age of the children in this group ranged between 10 and 36 months, with a median age of 20.3 months. Concerning gender, most children were male (n = 14; 70%) (Table 1). As for the results obtained, there was an agreement of results in 18 pairs of samples of which 17 were positive and 1 negative, that is, 90% of the plasma/saliva pairs showed the same result for TTV DNA. The results were discordant in 2 of the samples (TD224 and TD247). Both plasma samples were positive, but the saliva samples were negative. In these cases, the viral loads in plasma were the 2 lowest, 2.85 log_10_ copies/mL and 3.24 log_10_ copies/mL, respectively.

**Table 1.** Results Obtained in Real-Time PCR: Viral Load in Plasma and Saliva Samples.

Sample	Gender	Age, mo	Plasma (log_10_ copies/mL)	Saliva(log_10_ copies/mL)
TD244	M	15	NEG	NEG
TD247	M	15	2.85	NEG
TD224	M	19	3.24	NEG
TD242	F	24	3.72	1.49
TD239	M	19	4.12	3.48
TD241	M	24	4.15	4.04
TD231	M	28	4.24	2.47
TD219	M	36	4.28	3.33
TD234	F	19	4.37	4.23
TD233	M	17	4.42	3.67
TD246	F	19	4.43	3.52
TD232	M	13	4.57	4.21
TD217	M	14	4.60	2.62
TD237	F	25	4.81	4.62
TD218	M	28	4.84	6.19
TD238	M	22	4.98	4.61
TD235	F	16	5.03	3.56
TD240	F	23	5.07	3.30
TD245	M	10	5.28	5.88
TD236	M	18	5.30	3.64

M: male; F: female.

In most concordant pairs (16/18), the viral load was higher in plasma, only 2 samples showed higher viral loads in saliva (TD218 and TD245). Viral loads in plasma presented a median of 4.43 log_10_ copies/mL (confidence interval [95% level]: 3.713-4.717). In saliva, the median was 3.54 log_10_ copies/mL (confidence interval [95% level]: 2.496-3.990).

**Figure 2.**
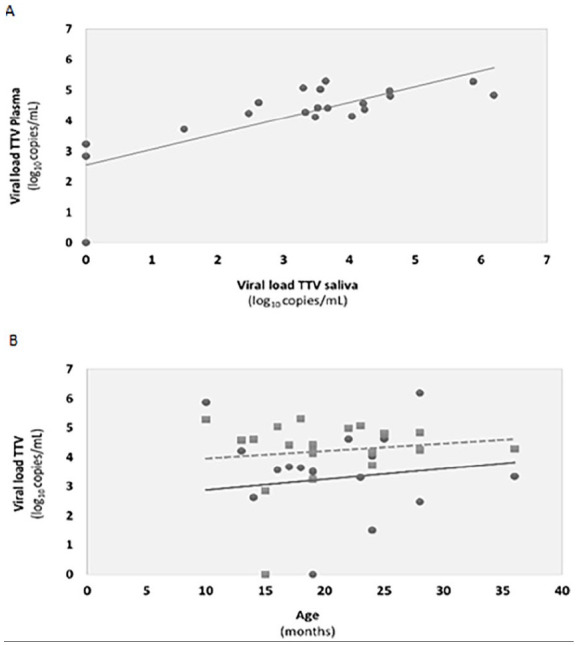
Correlations between (A) plasma and saliva viral loads determined by the Pearson test (*r* = .76; *P* < .0001); (B) plasma viral loads (squares; *r* = .132, *P* = .579) and saliva viral loads (dots; *r* = .128, *P* = .591) with age of patients. Tendency lines for plasma and saliva are represented with, respectively, dashed, and full lines.

A statistical correlation (Pearson test: *r* = .76; *P* < .0001) was found between the TTV viral load obtained in the plasma and saliva samples (Figure 2A). Regarding age, there was no correlation with either plasma or saliva viral loads (Figure 2B).

## Discussion

The results of our study detected a high prevalence of TTV infection in a Portuguese pediatric population aged 1 to 3 years, with an overall prevalence of 75.5%. The viral loads of saliva samples from these children were often high suggesting that saliva may be an important means of transmission of the virus. Previous studies done in children indicate that TTV is acquired early in childhood and that the prevalence increases with age.^2^ In a Finnish study, the prevalence increases from 39% at ^4^ months of age to 99 and 100% at 2 and 4 years of age, respectively.^14^ Despite some variability between studies, high prevalence is also observed in Japanese studies, ranging from 43% to 100% at 2 years of age,^7,9^ which suggests that these high rates are observed worldwide. Little is known, however, about the possibility of reinfection by this virus in pediatric ages, and what role, if any, genotypes may play in these possible reinfections.

Prenatal mother-to-child transmission is a controversial issue. Torque Teno virus was not detected in cord blood samples in 2 studies,^8,9^ or in amniotic fluid.^15^ On the contrary, 1 team detected TTV in 13.8% of 138 cord blood samples analyzed,^16^ and another author described viremia at birth in 2 neonates.^17^ An interesting study found that TTV DNA was not detected in the cord blood samples (0/160) using one primer set but could be detected in 11.8% (7/76) of samples using another set of primers.^11^ Therefore, a possible explanation for the different findings between studies could be the sensitivities of the different PCR techniques. The use of primers targeting more conserved regions of the viral genome may have an important contribution to the sensitivity of the PCR and to the data about TTV. Therefore, in the current research, selected primers were directed to the UTR region, for which both sensitivity and specificity had been previously demonstrated.^18^ Thus, we believe that the high rate of TTV in our survey accurately estimates the true prevalence of TTV in the study populations.

In our study, only 5 positive samples were detected out of a total of 138 neonates, representing a TTV prevalence of 3.7%. Compared with viral loads of children aged 1 to 3 years, neonates had lower loads.

The current study aimed to assess the prevalence of TTV in children younger than 3 years old, not the transmission mechanism, and so our protocol did not include maternal samples, cord blood samples, or neonate blood (collected in the first few days of life). Therefore, it was not possible to determine the origin of the positive cases in neonates, whether they were true cases acquired before or after birth, or whether they resulted from maternal contamination.

Despite this disadvantage of using saliva for neonates, there has been a growing interest in the use of this sample, especially due to the simplicity of the collection procedure and its noninvasive nature, which is critical in the pediatric population.^19^ These advantages allow its use in more extensive and longitudinal studies. These investigations will be crucial to better understanding TTV’s role in human health and disease. In our comparative analysis, there was a 90% agreement in the results of the paired saliva and plasma samples tested, suggesting that the virus can be present in different compartments. Considering the viral load of the samples, we also found a correlation between viral loads in plasma and saliva, although viral load was generally slightly higher and more stable in plasma. This was evident in the 2 negative saliva samples, which corresponded to the 2 plasma samples with the lowest viral loads. The greater variability with saliva samples, already described by others,^6^ may be due, in some cases, to the eventual contamination by mother’s milk, as previously mentioned. Interestingly, in adult patients with comorbidities, comparing TTV viral load of both saliva and plasma has given discordant results, that is, higher and lower saliva viral loads were described when compared with plasma viral loads.^20,21^

There was no correlation between age and viral load in this study, either in plasma or in saliva. The literature reports that, in general, the peak of infection occurs between 3 and 6 months, with great viral proliferation in the first 60 days, after which the viral load stabilizes.^2^ The average age of our study population (20.3 months) was much higher, and probably most of the children were picked up after the stabilization of the viral load.

The laboratory turnaround time for plasma or salivary viral loads can be adjusted, allowing a rapid response when necessary. Although these determinations are usually done in a research context and not routinely, their use as a potential biomarker for the degree of immunosuppression has been suggested.^22^

This study has some limitations, so care must be taken when extrapolating to the general population.

These limitations include the small sample size of the group comparing plasma and saliva samples. Given the scarcity of studies with saliva and plasma samples for TTV detection in pediatric patients, we performed this comparison as an exploratory study. Despite the low sample size, the results of this comparison are promising and add information, although they require larger studies validating saliva samples for epidemiological studies for TTV. The study does not represent the entire pediatric age group, and therefore, we cannot extrapolate the results beyond 3 years of age.

The samples from children 1 to 3 years old were collected at the pediatric emergency department of the hospital. Although the relationship between immunosuppression and viral load caused by TTV is well known, it is not expected that an acute illness will significantly influence the detection rate of this virus. However, we cannot exclude possible interferences of ongoing infections or other changes in the immune system that, somehow, may be reflected in the observed viral loads.

Another limitation of this study was the lack of quantification of total cellular DNA in saliva, making it impossible to infer the quality of saliva. The saliva samples used in the present study were collected with a swab and may contain nonuniform amounts of cells, so the viral load determined may be undervalued in some samples, hence the need for further standardization.

In the neonate population, we cannot conclude that the positive samples for TTV (3.6%) are cases of congenital infection. Therefore, more extensive studies in neonates are needed, considering additional factors such as the mother’s infection status and the TTV detection in the neonate’s blood.

There was a gap of several years between the completion of the project and the publication of the results, because of the COVID-19 pandemic. It is not known what impact, if any, the pandemic may have had on the prevalence of TTV in children, given the reduced contact between children during the pandemic. Therefore, the prevalence results presented here may not be exactly the current reality.

In conclusion, to the best of our knowledge, the present study was the first done in Portugal on the prevalence of TTV in a pediatric population. We found a correlation between TTV viral loads in plasma and saliva and a high prevalence (75.5%) of TTV in saliva in the studied pediatric population aged between 1 and 3 years. Studies with larger sampling to compare the plasma and saliva viral loads in children without acute situations are needed.

## Author Contributions

CB carried out the experiments and analyzed the data. PeP made data interpretations and wrote the manuscript. MJC designed the study, verified the data, and revised the manuscript. MT and MM collected the samples and made data interpretations. PaP designed the study, made data interpretations, and revised the manuscript. All authors contributed to the article and approved the submitted version.
